# IFITM1 and IFITM3 cooperate to restrict virus entry in endolysosomes

**DOI:** 10.1128/jvi.00677-26

**Published:** 2026-06-09

**Authors:** Isaiah Wilt, Abigail A. Jolley, Kazi Rahman, Kin Kui Lai, Mahesh Agarwal, Guoli Shi, Thorkell Andresson, Alex A. Compton

**Affiliations:** 1 Center for Cancer Research, National Cancer Institute, Frederick, Maryland, USA; 2 Frederick National Laboratory for Cancer Research 437329 , Frederick, Maryland, USA; Loyola University Chicago - Health Sciences Campus, Maywood, Illinois, USA

**Keywords:** innate immunity, IFITM, interferons, membrane transport, RNA virus, influenza

## Abstract

Interferon-induced transmembrane (IFITM) proteins restrict virus infections at the stage of cellular entry. We previously characterized a GxxxG motif in the CD225 domain of human IFITM3 that mediates its multimerization, which is essential for the reduction of membrane fluidity by IFITM3 and for its antiviral activity against the influenza A virus. Here, using an unbiased approach coupling immunoprecipitation with mass spectrometry, we show that the GxxxG motif is also important for the interaction of IFITM3 with other proteins, including IFITM1. IFITM1 is primarily regarded as a cell surface protein that restricts the entry of viruses fusing at the plasma membrane, but this model is based mostly on overexpression studies and is at odds with some studies showing that it can restrict endocytic viruses. Here, we show that endogenous IFITM1 and IFITM3 co-reside in membranes of acidic late endosomes and lysosomes and interact, as determined by co-immunoprecipitation and proximity ligation assay. Knockdown of endogenous IFITM3 resulted in enhanced localization of IFITM1 to the plasma membrane, indicating that IFITM3 promotes IFITM1 localization to endolysosomes. To assess the antiviral protection conferred by endogenous IFITM1 and IFITM3 against viruses fusing at endolysosomal membranes, we measured cellular infection by influenza A virus. While individual knockdown of IFITM1 or IFITM3 significantly increased infection, combined knockdown of both IFITM1 and IFITM3 boosted infection to a much greater extent. These results suggest that endogenous IFITM1 and IFITM3 restrict Influenza A virus entry in endolysosomes in a cooperative manner.

**IMPORTANCE:**

There exist multiple IFITM proteins encoded in the human genome, and the IFITM locus has expanded by gene duplication in multiple species for reasons that are poorly understood. Here, we show that two human IFITM proteins known for performing antiviral roles during virus infection, IFITM1 and IFITM3, interact with one another in endolysosomes. Using RNA interference, we found that knockdown of IFITM1 or IFITM3 resulted in elevated susceptibility to Influenza A virus infection. However, combined knockdown of both IFITM1 and IFITM3 led to even greater infection. These results demonstrate that both IFITM1 and IFITM3 act as barriers to Influenza A virus entry in endolysosomes. Furthermore, we found that IFITM3 is necessary for the trafficking of IFITM1 to endolysosomes. Overall, we reveal that IFITM proteins, which are related to one another through gene duplication, evolved to work together to restrict virus infection.

## INTRODUCTION

Prior to the recruitment of innate and adaptive immune cells to sites of virus infection, the cell-intrinsic or cell-autonomous immune response is the first line of defense restricting infection within individual cells and inhibiting the spread of virus to neighboring bystander cells (1). Cell-intrinsic immunity is mediated by proteins encoded by interferon (IFN)-stimulated genes, many of which target different steps of the virus replication cycle (2). Several hundred IFN-stimulated genes are upregulated in response to type I, type II, and type III interferons, and only a fraction of these have been assigned functions or are understood on a mechanistic level (3). One group of IFN-stimulated genes that have been identified as broad-spectrum inhibitors of virus infection are the interferon-induced transmembrane (IFITM) proteins, consisting of IFITM1, IFITM2, and IFITM3 in humans. Primarily restricting the step of cellular entry, this group of restriction factors is believed to reduce membrane fusion between virus and cell by altering membrane fluidity and curvature (4, 5). Of the human IFITM proteins, IFITM3 exhibits potent antiviral activity against viruses that undergo membrane fusion in endosomes (6, 7), including the influenza A virus (8–11). Furthermore, the physiological importance of IFITM3 *in vivo* has been demonstrated by increased viral disease severity in influenza-infected mice deficient in *Ifitm3* (12, 13) and by increased disease severity in influenza-infected humans carrying single-nucleotide polymorphisms in *IFITM3* (14–19). As a result, IFITM3 is the best-characterized member of the IFITM family, and its antiviral mechanism of action has been studied in greater detail.

IFITM1, IFITM2, and IFITM3 are paralogs that likely arose following multiple gene duplication events over evolutionary time, with IFITM3 serving as the most ancient, ancestral locus (20–22). Many studies have shown that IFITM proteins exhibit differences in subcellular localization. For example, some reports have suggested that IFITM1 is found primarily on the cell surface, while IFITM2 and IFITM3 are found primarily in early and late endosomes (10, 21, 23–29). It has been reported that viruses that enter cells by fusing at the plasma membrane, including respiratory syncytial virus, metapneumovirus, herpes simplex virus 1, and HIV-1, are restricted by IFITM1, and this has been attributed to its putative localization to the plasma membrane (30–32). However, other studies have reported that IFITM1 partially resides in intracellular membranes (31, 33–35) and that IFITM1 can inhibit virus entry taking place at sites other than the plasma membrane. For example, two studies have shown that IFITM1 exhibits antiviral activity against influenza A virus, which is known to fuse with endosomal membranes in a pH-dependent manner (8, 36). Furthermore, IFITM1 restricts the filoviruses Ebola virus and Marburg virus, which enter cells by fusing with lysosomal membranes (28). Therefore, the apparent or assumed subcellular localization of IFITM1 in cells cannot fully explain virus sensitivity to IFITM1-mediated restriction. Moreover, most of the work characterizing the properties of IFITM1 employs overexpression approaches in transformed human cell lines, often with an epitope tag, or even overexpression in non-human cells (33). The localization of endogenous IFITM1 protein that is constitutively expressed or interferon-upregulated in human cells, as well as how it relates to antiviral activity, has not been addressed. Moreover, it is unknown whether endogenous IFITM proteins interact with one another and whether these interactions *in situ* regulate their antiviral functions. While it was previously demonstrated that ectopic IFITM proteins could co-immunoprecipitate with one another (37), it was not addressed whether endogenous IFITM proteins interact and, if so, where these interactions occur on the subcellular level.

We previously found that IFITM3 forms homomultimers through a GxxxG motif found in its CD225 domain and that this motif is essential for the antiviral functions of IFITM3 (38). Since the GxxxG motif is conserved in IFITM1 and IFITM2, it is likely that IFITM1 and IFITM2 form homomultimers as well and that multimerization may be important for their respective functions. However, it remained a possibility that the GxxxG motif present in IFITM proteins enables interactions with other cellular proteins, including between different IFITM proteins. Here, we identified endogenous IFITM1 as a binding partner of IFITM3. Furthermore, we show that this interaction is dependent on the glycine-95 motif of IFITM3, indicating that the conserved GxxxG motif is important for mediating both homo- and heteromultimerization among IFITM family members. By examining the interaction between endogenous IFITM3 and endogenous IFITM1 in diverse cell types, we found that these two proteins interact in acidic late endosomes and lysosomes (endolysosomes). Intriguingly, silencing of endogenous IFITM3 resulted in an accumulation of IFITM1 at the plasma membrane, revealing IFITM3 as an important regulator of IFITM1 localization. Therefore, the presence of IFITM3 may influence the antiviral properties of IFITM1 in the same cell. Since we found endogenous IFITM1 and IFITM3 to co-reside in endolysosomal membranes, we measured the extent to which silencing of IFITM3 alone or IFITM1 and IFITM3 together impacted cellular entry mediated by influenza A hemagglutinin. Our results demonstrated that both endogenous IFITM1 and IFITM3 act as barriers to the endocytic entry of Influenza A virus. These results provide insight into the cooperative roles played by IFITM proteins in cellular endomembranes. Moreover, they suggest that experiments involving the overexpression or silencing of individual IFITM genes should be interpreted cautiously.

## RESULTS

**Fig 1:**
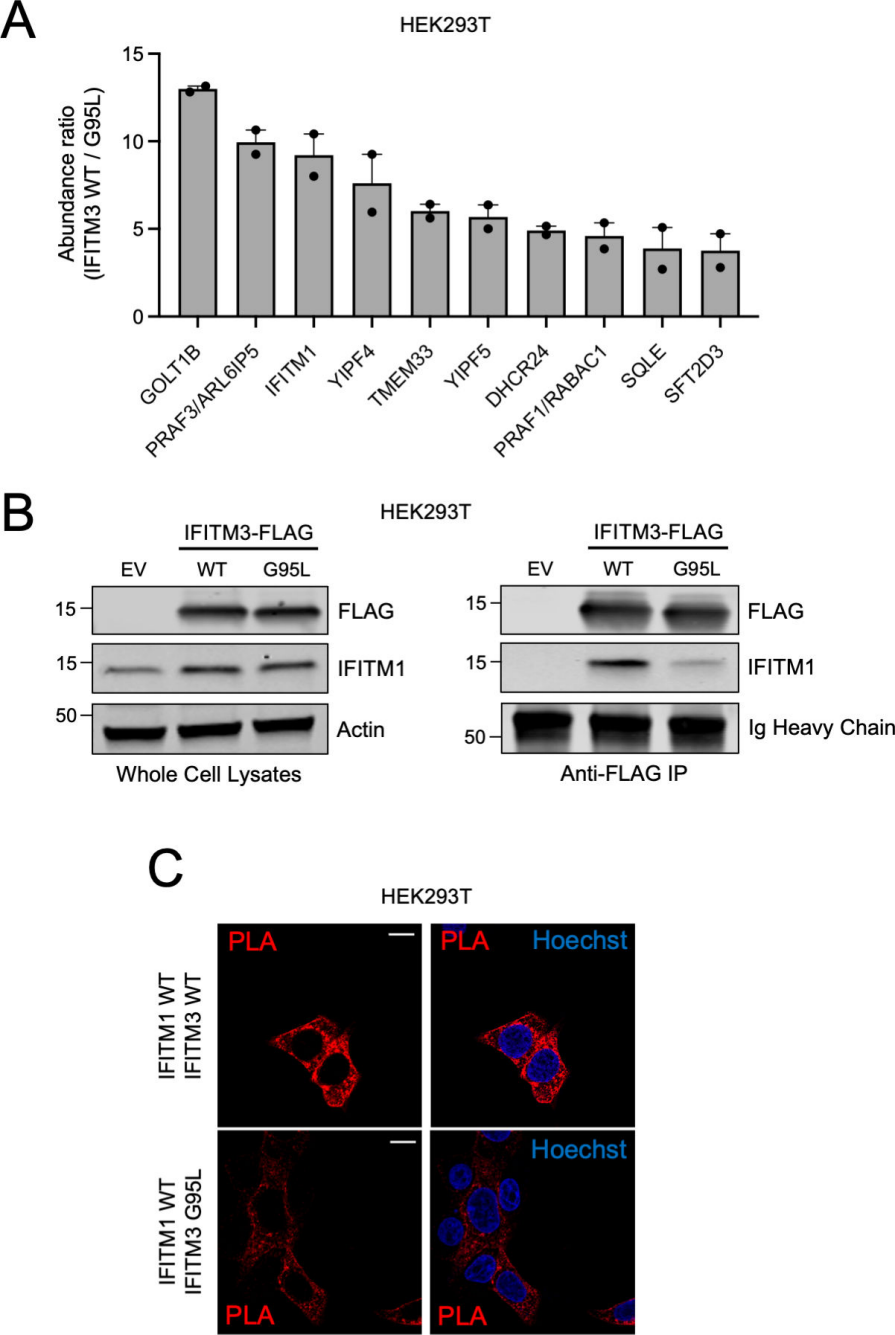
IFITM3 interacts with endogenous IFITM1 in a G95-dependent manner. (**A**) HEK293T cells stably expressing pQCXIP-FLAG-IFITM3 WT or pQCXIP-FLAG-IFITM3 G95L or pQCXIP-empty vector were lysed, and anti-FLAG immunoprecipitation was performed. Co-immunoprecipitated proteins were identified by mass spectrometry (MS). Abundance ratios were calculated, whereby total abundance from immunoprecipitated FLAG-IFITM3 WT was divided by total abundance from FLAG-IFITM3 G95L. The mean abundance ratio plus standard error is shown for a subset of proteins that pulled down to a greater extent with IFITM3 WT relative to IFITM3 G95L. Symbols represent ratios obtained from duplicate MS runs. (**B**) (Left) HEK293T cells stably expressing pQCXIP-FLAG-IFITM3 WT or pQCXIP-FLAG-IFITM3 G95L or pQCXIP-empty vector were lysed, and whole cell lysates were subjected to SDS-PAGE and immunoblotting with anti-FLAG, anti-IFITM1, and anti-actin (used as a loading control). (Right) FLAG-IFITM3 was immunoprecipitated with anti-FLAG, and IP fractions were subjected to SDS-PAGE and immunoblotting with anti-FLAG and anti-IFITM1 (immunoglobulin heavy chain served as a loading control). Numbers and tick marks left of blots indicate the position and size (in kilodalton) of protein standards in the ladder. (**C**) HEK293T cells were transiently transfected with 0.50 μg pQCXIP-FLAG-IFITM1 WT and 0.05 μg pQCXIP-FLAG-IFITM3 WT or pQCXIP-FLAG-IFITM3 G95L, fixed and permeabilized, and a proximity ligation assay was performed using anti-IFITM1 and anti-IFITM3 followed by confocal immunofluorescence microscopy. Nuclei were labeled with Hoechst. Scale bar = 15 microns. All immunoblots and microscopy experiments were performed twice, and one representative example is shown. EV, empty vector; Ig, immunoglobulin; PLA, proximity ligation assay; WT, wild type.

To identify interaction partners of IFITM3, including those dependent on the GxxxG motif of IFITM3 mediating homomultimerization and antiviral activity, we stably expressed FLAG-IFITM3 WT, FLAG-IFITM3 G95L, or empty vector in HEK293T cells, immunoprecipitated ectopic IFITM3 with an anti-FLAG antibody from whole cell lysates, and identified co-immunoprecipitated proteins with proteomics (File S1). Putative interactors of IFITM3 were qualified as those whose peptides were identified in the IFITM3 WT or IFITM3 G95L fractions or both but which were absent from the empty vector fraction. Furthermore, we sorted interactors that associated with IFITM3 WT to a greater extent than IFITM3 G95L to identify protein-protein interactions that depended on the presence of glycine-95. For each protein, overall abundance pulled down by IFITM3 WT was divided by abundance pulled down by IFITM3 G95L to derive an abundance ratio. Among the partners that preferentially pulled down with FLAG-IFITM3 WT was IFITM1 (Fig. 1A). We found that IFITM3 WT pulled down approximately ninefold more endogenous IFITM1 compared to IFITM3 G95L, indicating that G95 of IFITM3 promotes the interaction between IFITM3 and IFITM1 (Fig. 1A).

To verify that endogenous IFITM1 interacts with ectopic FLAG-IFITM3 in a G95-dependent manner, FLAG-IFITM3 WT and FLAG-IFITM3 G95L were immunoprecipitated with anti-FLAG, and pull-down of endogenous IFITM1 was determined by immunoblotting with an anti-IFITM1 antibody. The specificity of anti-IFITM1 was determined by immunoblotting lysates from HeLa and HeLa IFITM3 knockout cells treated with type I interferon or not (Fig. S1A). We found that endogenous IFITM1 pulled down with IFITM3 WT to a greater extent than with IFITM3 G95L, validating our proteomics result (Fig. 1B). To determine whether IFITM3 and IFITM1 interact at the plasma membrane, intracellular membranes, or both, we performed a proximity ligation assay (PLA) and confocal immunofluorescence microscopy using anti-IFITM3 and anti-IFITM1 in HEK293T transfected with IFITM1 and IFITM3. We found that PLA fluorescence was detected at or near the plasma membrane and was especially apparent in intracellular compartments in cells transfected with IFITM1 and IFITM3 WT. In contrast, fluorescence was significantly decreased in cells transfected with IFITM1 and IFITM3 G95L (Fig. 1C). These results suggest that overexpressed IFITM3 interacts with both endogenous and overexpressed IFITM1 in a G95-dependent manner, and that this interaction can occur within endomembranes of cells.

**Fig 2:**
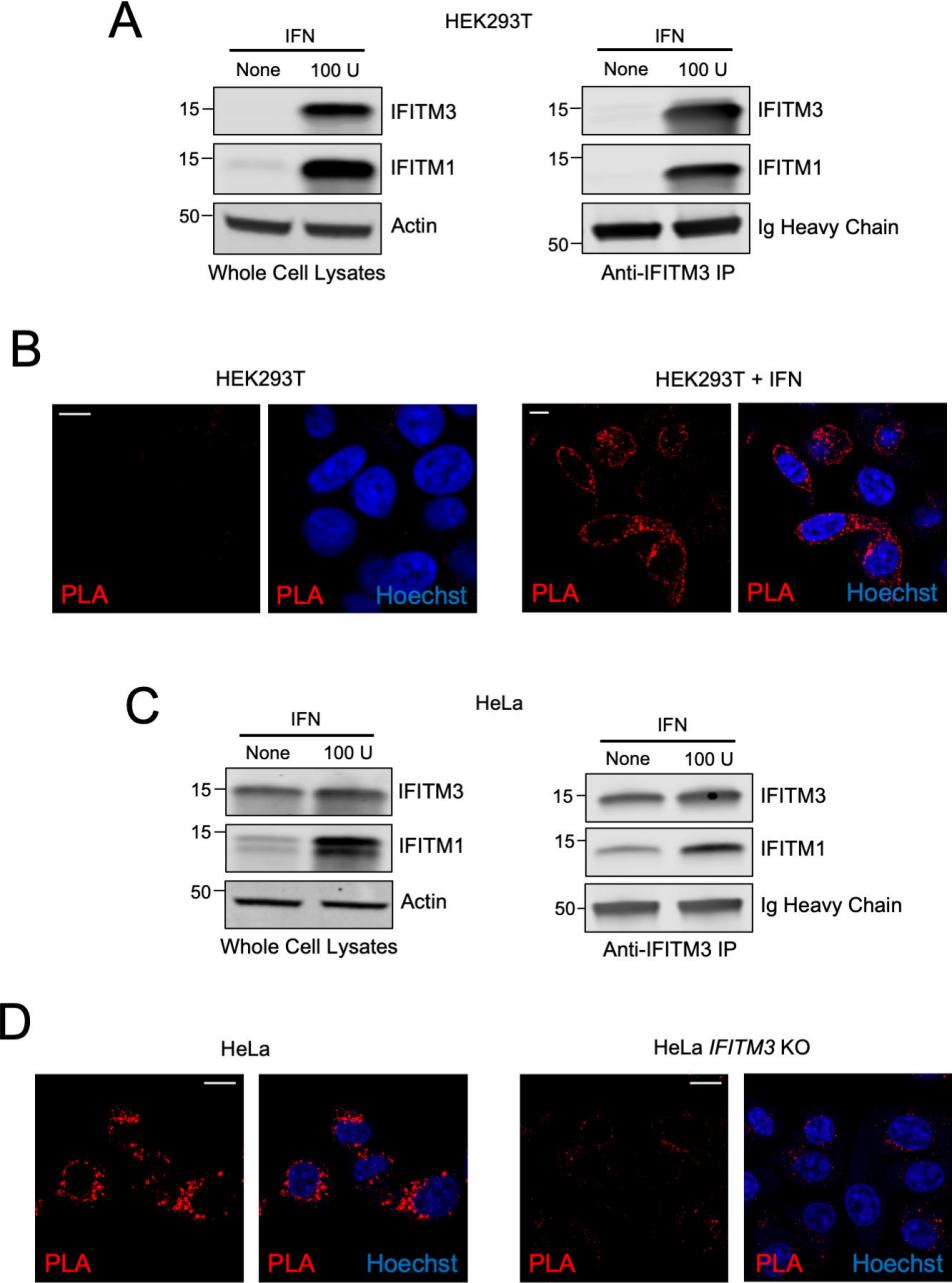
Endogenous IFITM3 and IFITM1 interact in multiple cell types. (**A**) (Left) HEK293T cells were untreated or treated with 100 units of type I interferon (IFNb) for 18 h, and whole cell lysates were subjected to SDS-PAGE and immunoblotting with anti-IFITM3, anti-IFITM1, and anti-actin (used as a loading control). (Right) IFITM3 was immunoprecipitated with anti-IFITM3, and IP fractions were subjected to SDS-PAGE and immunoblotting with anti-IFITM3 and anti-IFITM1 (immunoglobulin heavy chain served as a loading control). (**B**) HEK293T cells were untreated or treated with 100 units of IFNb for 18 h, fixed and permeabilized, and subjected to proximity ligation assay using anti-IFITM1 and anti-IFITM3, followed by confocal immunofluorescence microscopy. Nuclei were labeled with Hoechst. Scale bar = 10 microns. (**C**) (Left) HeLa cells were untreated or treated with 100 units of IFNb for 18 h, and whole cell lysates were subjected to SDS-PAGE and immunoblotting with anti-IFITM3, anti-IFITM1, and anti-actin (used as a loading control). (Right) IFITM3 was immunoprecipitated with anti-IFITM3, and IP fractions were subjected to SDS-PAGE and immunoblotting with anti-IFITM3 and anti-IFITM1 (immunoglobulin heavy chain was used as a loading control). Numbers and tick marks left of blots indicate the position and size (in kilodalton) of protein standards in the ladder. (**D**) HeLa and HeLa IFITM3 knockout cells were fixed and permeabilized, and proximity ligation assay was performed using anti-IFITM1 and anti-IFITM3, followed by confocal immunofluorescence microscopy. Nuclei were labeled with Hoechst. Scale bar = 15 microns. All immunoblots and microscopy experiments were performed twice, and one representative example is shown. IFN, interferon; KO, knockout; U, units.

While our findings reveal that IFITM1 and IFITM3 can interact with one another when one or both proteins are overexpressed in HEK293T cells, we next assessed the degree to which endogenously expressed IFITM1 and IFITM3 associate with each other when they are expressed constitutively or upregulated following interferon treatment. In HEK293T cells, the basal level of IFITM3 was undetectable, while a limited quantity of IFITM1 was observed; both IFITM1 and IFITM3 were significantly upregulated by type-I interferon (Fig. 2A). When IFITM3 was immunoprecipitated from interferon-treated cells, we found that IFITM1 co-immunoprecipitated with it (Fig. 2A). This interaction was validated in intact cells using PLA, which demonstrated that endogenous IFITM1 and IFITM3 interact, in a direct or indirect manner, throughout the cytosolic space in interferon-treated cells (Fig. 2B). We performed similar experiments in HeLa, since we and others previously demonstrated that IFITM proteins are constitutively expressed and restrict virus infections in these cells (39, 40). As expected, both IFITM1 and IFITM3 were observed in HeLa under basal conditions, with IFITM3 being more abundant (Fig. 2C). Here, we observed that IFITM1 pulled down with immunoprecipitated IFITM3 in the absence and presence of interferon (Fig. 2C). Confirming that IFITM1 and IFITM3 proteins interact constitutively in HeLa cells, abundant fluorescence was observed in perinuclear regions according to PLA, and this signal was abrogated in HeLa IFITM3 knockout cells (Fig. 2D). Since IFITM2 is a close paralog of IFITM3, and since it is constitutively expressed in HeLa (albeit to a lesser extent than IFITM3) (Fig. S1B), we immunoprecipitated IFITM2 using a specific anti-IFITM2 antibody. We observed that both IFITM1 and IFITM3 co-immunoprecipitated with IFITM2 in HeLa cells, especially in interferon-treated cells (Fig. S1B).

**Fig 3:**
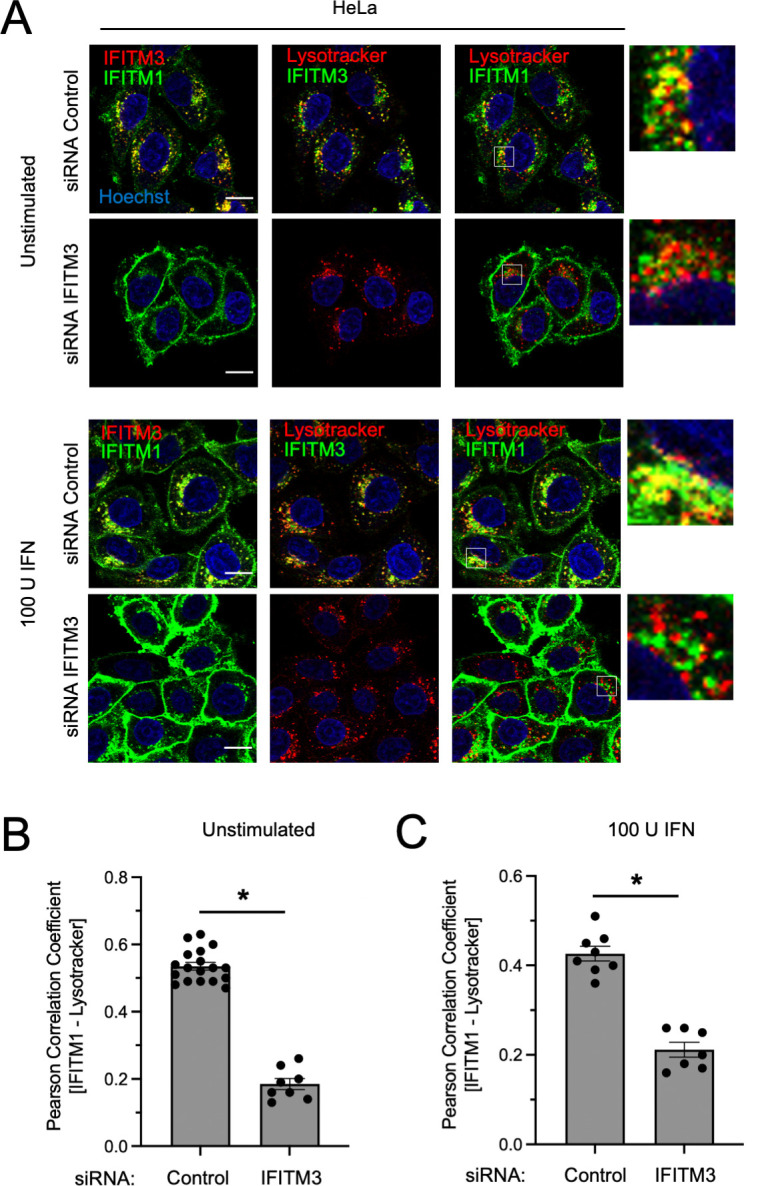
Endogenous IFITM1 and IFITM3 co-reside in endolysosomal membranes, and the localization of IFITM1 to endolysosomes is IFITM3 dependent. (**A**) HeLa cells were transfected with control siRNA or siRNA targeting IFITM3 for 48 h and subsequently treated with 100 units of IFNb1a for 18 h or left untreated. Cells were stained with LysoTracker, fixed and permeabilized, and immunostained with anti-IFITM1 and anti-IFITM3, followed by confocal immunofluorescence microscopy. Scale bar = 15 microns. (**B**) Co-localization between IFITM1 and LysoTracker in untreated cells was quantified by Pearson correlation coefficient and plotted as mean plus standard error. Symbols represent fields of view containing 5–12 cells. Differences that were statistically significant between the indicated conditions as determined by Student’s *t*-test are indicated by * (*P* < 0.05). (**C**) Co-localization between IFITM1 and LysoTracker in cells treated with 100 units of IFNb1a was quantified by Pearson correlation coefficient and plotted as mean plus standard error. Symbols represent fields of view containing 5–12 cells. Differences that were statistically significant between the indicated conditions as determined by Student’s *t*-test are indicated by * (*P* < 0.05). All microscopy experiments were performed twice, and one representative example is shown.

**Fig 4:**
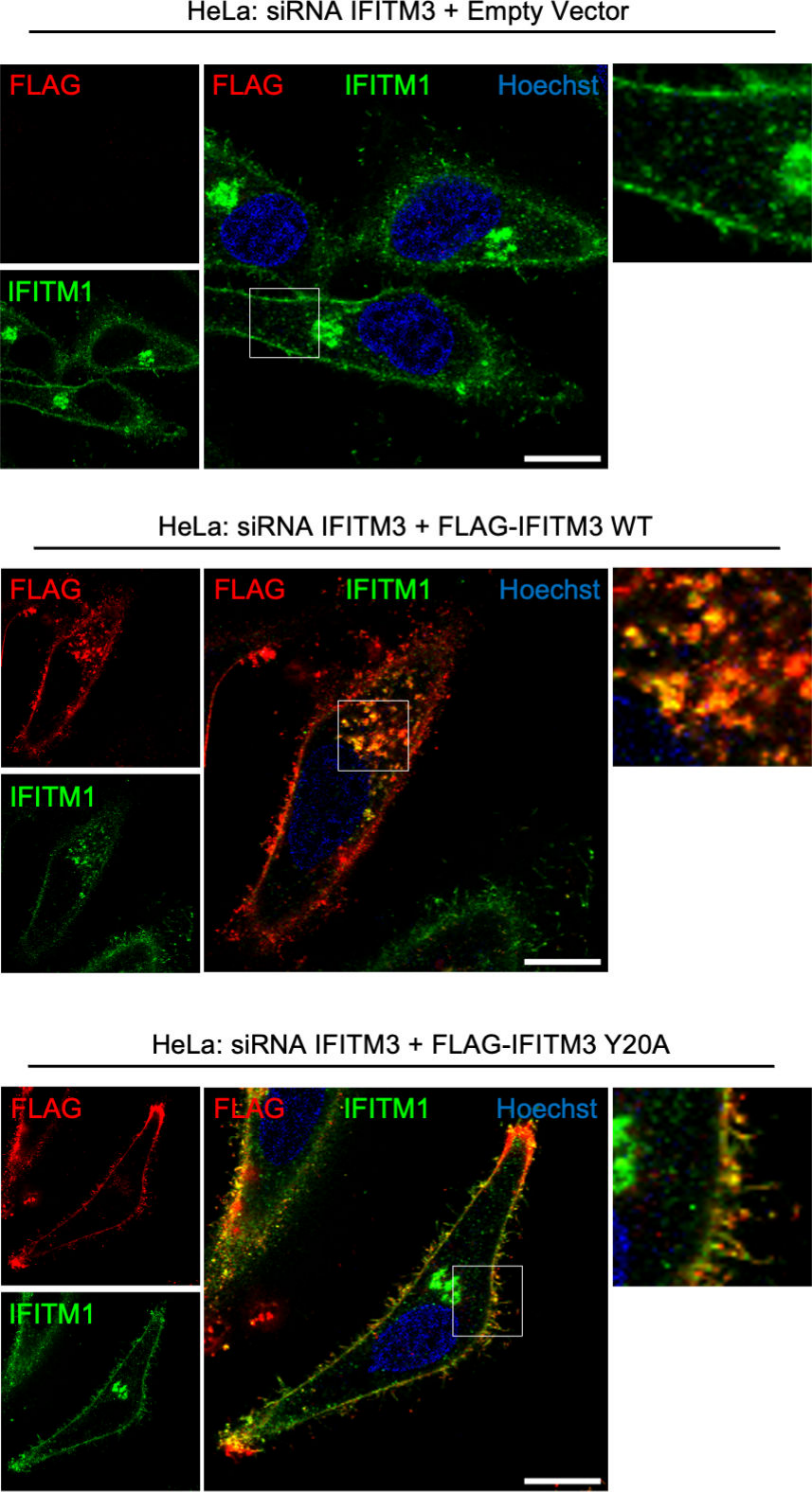
Complementation of IFITM3-deficient cells with IFITM3 WT restores IFITM1 intracellular localization in a Y20-dependent manner. HeLa cells were transfected with siRNA targeting IFITM3 for 48 h and subsequently transfected with 0.75 μg of pQCXIP-FLAG-IFITM3 (WT or Y20A) or an empty vector. Six hours later, media were removed and replaced with media containing 100 units of IFNb1a. Eighteen hours later, cells were fixed, permeabilized, and immunostained with anti-IFITM1 and anti-FLAG followed by confocal immunofluorescence microscopy. Nuclei were labeled with Hoechst. Scale bar = 15 microns. All microscopy experiments were performed twice, and one representative example is shown.

We then performed confocal immunofluorescence microscopy to characterize the subcellular localization of endogenous IFITM1 and IFITM3. Partial co-localization between IFITM1 and IFITM3 at vesicular sites was observed in HeLa cells, and this was also the case in interferon-treated cells, although an apparent increase in IFITM1 at the cell periphery was observed (Fig. 3A). In the same acquisitions, we assessed the position of IFITM1-positive vesicular compartments relative to LysoTracker, a marker of acidic late endosomes and lysosomes (endolysosomes). We found that a proportion of IFITM1 co-localized with LysoTracker in the absence and presence of interferons (Fig. 3B and C). A portion of IFITM3 was also co-localized with LysoTracker, and the same LysoTracker-positive compartments contained both IFITM1 and IFITM3 (Fig. 3A). Therefore, pools of IFITM1 and IFITM3 proteins co-localize in endolysosomes in HeLa cells at constitutive and interferon-induced levels. As a control to confirm the specificity of the anti-IFITM1 antibody, we knocked down endogenous IFITM3 with siRNA that selectively reduces IFITM3 protein. Surprisingly, while total IFITM1 levels were unaffected, there was an evident accumulation of IFITM1 at or near the cell periphery following knockdown of IFITM3 (Fig. 3A). Furthermore, the co-localization between IFITM1 and LysoTracker was significantly reduced in IFITM3 knockdown cells (Fig. 3B and C). When IFITM3 knockdown cells were transfected with ectopic IFITM3 WT, pools of endogenous IFITM1 were seen to co-localize with IFITM3 in intracellular compartments resembling endolysosomes (Fig. 4). However, ectopic expression of IFITM3 bearing the Y20A mutation, which disrupts the AP-2-dependent targeting of IFITM3 to the endocytic network (25–27), resulted in co-localization between IFITM1 and IFITM3 at or near the cell surface (Fig. 4). These findings suggest that IFITM3 promotes the localization of endogenous IFITM1 to acidic endolysosomes in a manner that requires the endocytic trafficking of IFITM3.

To determine whether endogenous IFITM1 and IFITM3 associate in primary epithelial cells, we examined primary human nasal epithelia. Both IFITM1 and IFITM3 were detectable at a constitutive level in primary epithelia; protein levels were enhanced following interferon treatment; and IFITM1 co-immunoprecipitated with IFITM3 in interferon-treated cells (Fig. S2A). Furthermore, co-localization between IFITM1 and IFITM3 was apparent in primary epithelia, especially following interferon treatment (Fig. S2B). As was observed in HeLa cells, knockdown of IFITM3 in primary epithelia resulted in increased abundance of IFITM1 at or near the cell surface (Fig. S2B). Therefore, our results suggest that endogenous IFITM1 and IFITM3 interact with one another in transformed and primary cells that are commonly used as targets for enveloped virus infection.

**Fig 5:**
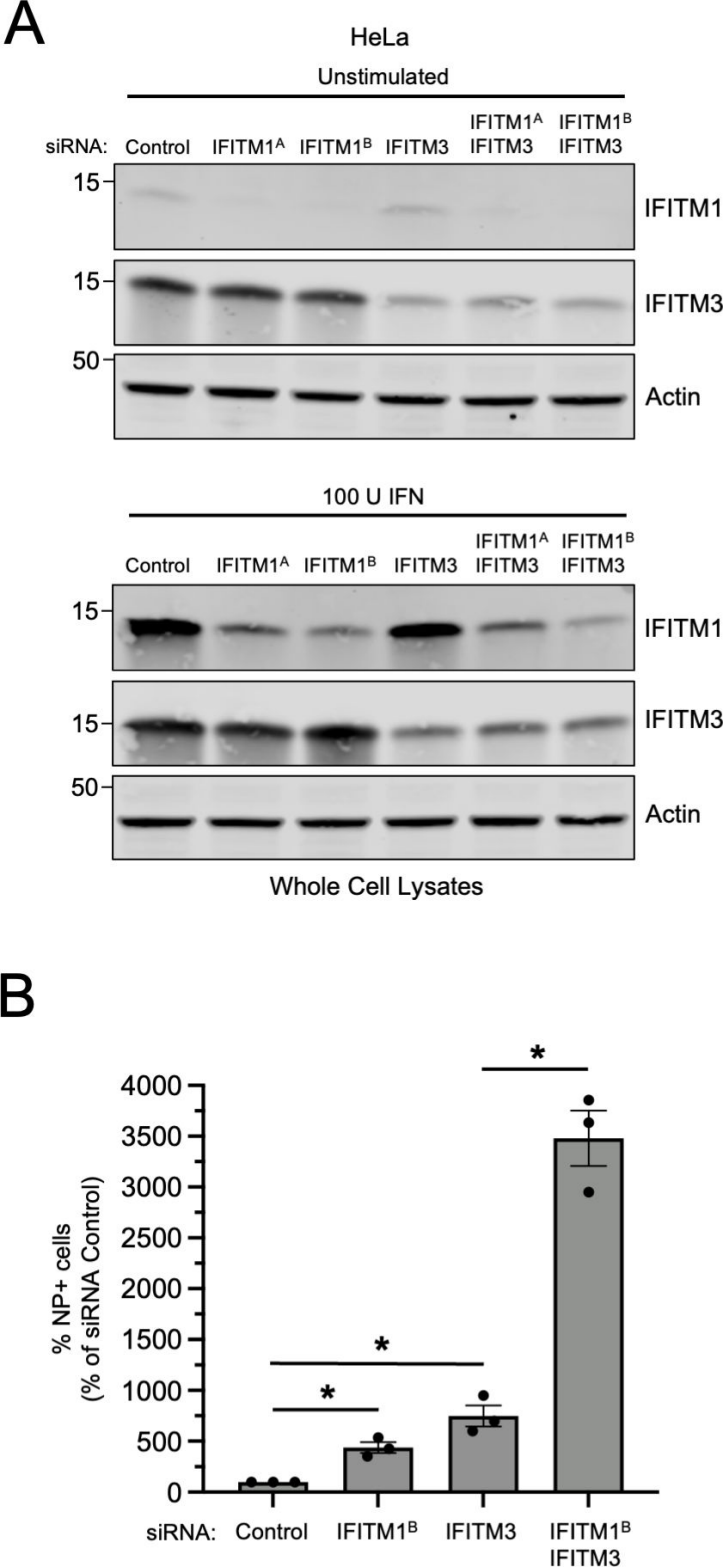
Endogenous IFITM1 and IFITM3 restrict influenza A virus infection. (**A**) HeLa cells, unstimulated or treated with type-I IFN (IFNb1a), were transfected with control siRNA, siRNA targeting IFITM1 (termed IFITM1^A^ or IFITM1^B^), siRNA targeting IFITM3, or siRNA targeting IFITM3 plus siRNA targeting IFITM1^A^ or IFITM1^B^ for 72 h. Cells were lysed, and whole cell lysates were subjected to SDS-PAGE and immunoblotting with anti-IFITM1, anti-IFITM3, and anti-actin (used as loading control). Numbers and tick marks left of blots indicate the position and size (in kilodaltons) of the protein standard in the ladder. Immunoblotting from two independent experiments was performed, and one representative example is shown. (**B**) HeLa cells were transfected with control siRNA, siRNA targeting IFITM1 (IFITM1^B^), siRNA targeting IFITM3, or siRNA targeting IFITM3 plus siRNA targeting IFITM1 (IFITM1^B^) for 72 h and inoculated with influenza A/PR/8/34 (H1N1) at an MOI of 0.1. Infection was scored by immunostaining with anti-NP antibody at 18 h post-inoculation and plotted as mean plus standard error. Symbols represent three independent experiments (biological replicates). Differences that were statistically significant between the indicated conditions as determined by Student’s *t*-test are indicated by * (*P* < 0.05). IFN, interferon.

Having found that endogenous IFITM1 and IFITM3 interact and co-localize in endolysosomes, we sought to measure the capacity of both proteins to restrict a well-characterized enveloped virus from entering/fusing in endolysosomes. siRNAs that specifically silenced either IFITM1 or IFITM3 in HeLa cells were identified, and knockdown specificity was confirmed in unstimulated and interferon-treated cells (Fig. 5A). Next, we assessed how individual or combined knockdown of IFITM1 and IFITM3 influenced susceptibility to influenza A virus infection. Of the two siRNAs that downmodulated IFITM1 without impacting the IFITM3 protein level, one resulted in more pronounced silencing (IFITM1^B^). siRNA-transfected cells were challenged with the influenza A virus, and virus infection was scored by immunostaining of viral NP protein and flow cytometry. Specific knockdown of IFITM1 using IFITM1^B^ resulted in a modest but significant boost to infection (4.5-fold relative to control siRNA), suggesting that endogenous IFITM1 constitutively expressed in cells performs antiviral activity against influenza A virus (Fig. 5B). In comparison, specific knockdown of IFITM3 had a slightly greater effect (7.5-fold). However, when IFITM1 and IFITM3 were downmodulated together, infection was boosted to a much greater extent (35-fold) (Fig. 5B). These results indicate that combined silencing of IFITM1 and IFITM3 results in increased cellular susceptibility to influenza A virus infection compared to silencing of IFITM1 or IFITM3 alone. Therefore, endogenous IFITM1, which co-localizes with endogenous IFITM3 in endolysosomes, contributes to the restriction of the influenza A virus. Together with our observations that IFITM3 controls the localization of IFITM1 to endolysosomes, we propose that IFITM1 and IFITM3 cooperate to restrict virus entry in this compartment.

## DISCUSSION

In this report, we show that endogenous IFITM1 associates with endogenous IFITM3 in multiple cell types, including primary human epithelial cells of the respiratory tract targeted by influenza A virus. The interaction between IFITM3 and IFITM1 seems to be consequential for IFITM1 function, since silencing of IFITM3 results in accumulation of IFITM1 at the plasma membrane. The observation that manipulation of IFITM3 levels can impact IFITM1 has important implications for the interpretation of past and future experiments that rely upon the overexpression or silencing of individual IFITM genes. Phenotypes generated by siRNA-mediated knockdown or CRISPR-Cas9-mediated knockout of IFITM3 may, in whole or in part, result from the indirect effect those manipulations have on the IFITM1 protein. For example, the increase in influenza A infection following IFITM3 knockdown may, at least in part, be due to the shift in IFITM1 localization to the plasma membrane. We show here that both endogenous IFITM1 and endogenous IFITM3 contribute to the restriction of influenza A virus entry since infection is boosted when they are knocked down individually and boosted even further when they are knocked down in combination. However, these results likely underestimate the antiviral role of endogenous IFITM1, since we also show that loss of endogenous IFITM3 results in relocalization of IFITM1 to the cell surface. One previous study showed that combined knockout of IFITM1, IFITM2, and IFITM3 in HeLa did not result in more elevated influenza A infection compared to IFITM2 and IFITM3 knockout alone (41), and this was interpreted as suggesting that IFITM1 does not have an antiviral role to play against influenza A virus infection in HeLa cells. However, the lack of additional effect resulting from IFITM1 knockout might be explained by the fact that IFITM1 is mislocalized in the IFITM2/3 knockout cells. Also, it is unknown how IFITM2, or the lack thereof, may influence the antiviral activity of IFITM1.

Our results imply that the localization and activities of IFITM1 are influenced by the relative abundance of IFITM3 in the same cell. Since the constitutive level of IFITM3 protein present in different cell types may vary, the localization and activity of IFITM1 may also vary between cell types as a result. Indeed, it has been shown that IFITM1 exhibits cell type-dependent effects during certain virus infections (6, 7, 28, 33). Furthermore, it is likely that interferons can impact IFITM1 localization and function not only by upregulating IFITM1 itself but also by upregulating IFITM3. In addition to the impact of IFITM3 on IFITM1 localization and function, it is also possible that IFITM1 contributes to phenotypes resulting from IFITM3 overexpression, since we show that overexpressed IFITM3 interacts with endogenous IFITM1 (Fig. 1B). These findings provide a new, expanded framework for interpreting the effects of single IFITM knockdown or single IFITM overexpression.

Our findings indicate that the interaction between IFITM3 and IFITM1 impacts the localization of IFITM1. In the presence of IFITM3 WT, which is bestowed with a YxxL motif that recruits AP-2 and promotes its endocytosis, IFITM1 can be observed in association with IFITM3 at the cell surface and in endolysosomes. However, when the YxxL motif of IFITM3 is mutated to interfere with IFITM3 endocytosis, IFITM1 resides primarily at or near the cell surface (Fig. 4). Therefore, it appears that a population of IFITM1 hitchhikes with IFITM3 toward endolysosomes in a manner that depends on the interaction between IFITM3 and AP-2. Nonetheless, other factors may contribute to the localization of IFITM1 in cells. It was previously shown that IFITM1 contains an endocytic sorting signal in its carboxy terminus, which enables AP-3-dependent targeting to endolysosomes (31, 34, 35). It is worth examining whether IFITM3 regulates the exposure or recognition of this sorting signal present in IFITM1. It is possible that, in the presence of IFITM3, the carboxy terminal sorting signal of IFITM1 is stabilized or otherwise made more accessible to AP-3.

We recently demonstrated that IFITM3 promotes the lysosomal delivery of endocytic cargo by regulating fusion between late endosomes and lysosomes, and this newfound function is functionally associated with the restriction of influenza A virus entry (42). This activity requires a SNARE-like motif in the CD225 domain of IFITM3 that is also present in IFITM1, IFITM2, and other members of the CD225 superfamily. Our findings presented here depict IFITM1 as an endolysosomal protein when IFITM3 is present, raising the possibility that IFITM1 is also capable of regulating SNARE proteins involved in endosome-lysosome fusion and may do so in cooperation with IFITM3 as a part of its antiviral duties.

IFITM2 is the product of a recent gene duplication of IFITM3 in the ancestor of Homininae (humans, chimpanzees, and gorillas) (21). Since we show that endogenous IFITM2 co-immunoprecipitates with both IFITM1 and IFITM3, it may provide an additional regulatory layer to the function of IFITM1. Future work will focus on determining whether IFITM2 and IFITM3 perform redundant or distinct roles with regard to regulating the subcellular localization of IFITM1. Overall, our results reveal how regulatory relationships arose between closely related antiviral effectors that originated through multiple gene duplication events. Since unique duplications of IFITM genes have occurred in many species outside of humans (21, 43), resulting in unique repertoires of IFITM proteins that vary by species, there likely exist diverse mechanisms of cooperativity and regulation between IFITM proteins that remain to be discovered.

## MATERIALS AND METHODS

### Cells and cell culture reagents

HEK293T (ATCC, CRL-3216) and HeLa (ATCC, CCL-2) were cultured in Dulbecco’s modified Eagle’s medium (DMEM, Gibco) supplemented with 10% heat-inactivated fetal bovine serum (Hyclone) and 1% penicillin-streptomycin (Gibco) at 37°C with 5% CO_2_. HeLa IFITM3 KO cells were previously described (42). HEK293T stably expressing pQCXIP-empty vector, pQCXIP-FLAG-IFITM3 WT, and pQCXIP-FLAG-IFITM3 G95L was previously described (38). Human primary nasal epithelial cells (Epithelix, EP51AB) were cultured in hAEC Culture Medium (Epithelix, EP09AM) at 37°C with 5% CO_2_. Human interferon beta 1a was obtained from PBL Assay Science (11415-1). LysoTracker Deep Red was obtained from Thermo Fisher (L12492).

### SDS-PAGE, Western blot analysis, and immunoprecipitation

Whole cell lysis was performed using a buffer consisting of 20 mM HEPES, 150 mM NaCl, 1 mM EDTA, and 1% Triton X-100 (Sigma, X100) containing Halt protease inhibitor cocktail, EDTA-free (Thermo Fisher, 78425). Lysis was performed on ice for 30 min prior to centrifugation at 12,000 rpm for 10 min at 4°C, and supernatants were harvested. Protein quantity was measured using the Protein Assay (Bio-Rad, 5000001). Lysates were mixed with NuPAGE Reducing Agent (Invitrogen, NP0009) and Protein Loading Buffer (Li-COR, 928-40004) and loaded into Criterion XT 12% polyacrylamide Bis-Tris gels (Bio-Rad, 3450117). SDS-PAGE was performed with NuPAGE MES SDS Running Buffer (Invitrogen, NP0002). Proteins were transferred to an Amersham Protein Nitrocellulose Membrane, with a pore size of 0.2 microns (GE Healthcare, 10600004).

The following primary antibodies were used: anti-IFITM3 antibody (Abcam, ab109429), anti-FLAG antibody (Sigma, F7425), anti-IFITM1 (Proteintech, 60074-1-Ig), and anti-IFITM2 (Proteintech, 66137-1-Ig), anti-actin (Santa Cruz Biotechnology, sc-47778). The following secondary antibodies were used: goat anti-mouse IRDye 800CW (Li-COR, 926-32210), goat anti-mouse IRDye 680RD (Li-COR, 926-68070), goat anti-rabbit 800CW (Li-COR, 926-32211), and goat anti-rabbit 680RD (Li-COR, 926-68071). For immunoprecipitation, anti-IFITM3 antibody (Abcam, ab109429) or anti-FLAG antibody (Sigma, F7425) was added to 300 μg whole cell lysates, and the protein-antibody mixture was incubated for 3 h at 4°C. Dynabeads Protein G (15 μL; Invitrogen, 10007D) (pre-washed in cell lysis buffer) was added to the protein-antibody mixture and incubated by rotating for 1 h at 4°C, and reaction mixtures were centrifuged at 1,000 × *g* for 3 min at 4°C. The supernatant was removed, and the pelleted beads were washed three times with cell lysis buffer. The bead fraction was resuspended with NuPAGE Reducing Agent (Invitrogen, NP0009) and Protein Loading Buffer (Li-COR, 928-40004), boiled for 5 min at 95°C, and loaded into Criterion XT 12% polyacrylamide Bis-Tris gels (Bio-Rad, 3450117). Images were obtained with the Li-COR Odyssey CLx, and analysis was performed with ImageStudio Lite software (Li-COR). PageRuler Prestained Protein Ladder (10–180 kDa) was used as a protein standard (Thermo Fisher, 26616).

### Confocal immunofluorescence microscopy

Cells were seeded in eight-well Mu chamber slides (Ibidi, 80826) at 15,000 cells per well. For HEK293T cells, chamber slides were coated with 3 mg/mL PureCol for 1 h at room temperature and allowed to dry prior to cell seeding. Living cells were stained with LysoTracker Deep Red (Thermo Fisher, L12492) at a final concentration of 50 nM in complete DMEM for 30 min at 37°C. Cells were washed with D-PBS (Gibco) and fixed/permeabilized with Cytofix/Cytoperm Solution (BD, 554722) for 10 min at room temperature. Cells were washed with Cyto Perm/Wash Buffer (BD, 554723) and blocked in Intercept Blocking Buffer (PBS) (Li-COR, 927-70001) for 1 h at room temperature. Primary antibodies (rabbit anti-IFITM3 (Abcam, ab109429), mouse anti-FLAG (M2) (Sigma, F1804), and mouse anti-IFITM1 (Proteintech, 60074-1-Ig) were diluted 200-fold in Intercept T20 Antibody Diluent (PBS) (Li-COR, 927-75001), and cells were incubated for 3 h at room temperature. Cells were washed twice with Cyto Perm/Wash Buffer. Secondary antibodies (goat anti-mouse IgG [H + L] secondary antibody, Alexa Fluor 488 [Invitrogen, A11001], and goat anti-rabbit IgG [H + L] secondary antibody, Alexa Fluor 555 [Invitrogen, A21428]) were diluted 300-fold in Intercept T20 Antibody Diluent, and cells were incubated for 1 h at room temperature. Cells were washed twice with Cyto Perm/Wash Buffer and stained with Hoechst 33342 (Thermo Fisher, H3570) diluted 3,000-fold in D-PBS for 5 min. Cells were washed with Cyto Perm/Wash Buffer and imaged in D-PBS. Image acquisition was performed with a Leica Stellaris confocal fluorescence microscope. Images were analyzed and processed using Fiji (Image J).

### Immunoprecipitation-mass spectrometry

HEK293T stably expressing pQCXIP-FLAG-IFITM3 WT, pQCXIP-FLAG-IFITM3 G95L, or pQCXIP-empty vector were lysed (38), and immunoprecipitation of FLAG-IFITM3 was performed with anti-FLAG (M2) (Sigma, F1804) according to the protocol indicated above.

#### Protein digestion and TMT labeling

Immunoprecipitated fractions were lysed in 50 mM HEPES, pH 8.0, and 8 M urea, followed by sonication. Lysates were clarified by centrifugation, and protein concentration was quantified using a BCA protein estimation kit (Thermo Fisher). Lysate (100 μg) was alkylated and digested by the addition of trypsin at a ratio of 1:50 (Promega) and incubating overnight at 37°C. Digestion was acidified by adding formic acid (FA) to a final concentration of 1% and desalted using peptide desalting columns (Thermo Fisher) according to the manufacturer’s protocol. Peptides were eluted from the columns using 50% ACN/0.1% FA, dried in a SpeedVac, and kept frozen at −20°C until further analysis. For TMT labeling, 15 μg of each sample was reconstituted in 50 μL of 50 mM HEPES (pH 8.0), and 75 μg of TMTpro label (Thermo Fisher) in 100% ACN was added to each sample. After incubating the mixture for 1 h at room temperature with occasional mixing, the reaction was terminated by adding 8 μL of 5% hydroxylamine. The peptide samples for each condition were pooled and cleaned using peptide desalting columns (Thermo Fisher).

#### High pH reverse-phase fractionation

The first dimensional separation of the peptides was performed using a Waters Acquity UPLC System coupled with a fluorescence detector (Waters) using a 150 mm × 3.0 mm Xbridge Peptide BEM 2.5 μm C18 column (Waters) operating at 0.35 mL/min. The dried peptides were reconstituted in 100 μL of mobile phase A solvent (3 mM ammonium bicarbonate, pH 8.0). Mobile phase B was 100% acetonitrile (Thermo Fisher). The column was washed with mobile phase A for 10 min followed by gradient elution 0%–50% B (10–60 min) and 50%–75% B (60–70 min). The fractions were collected every minute. These 60 fractions were pooled into 24 fractions. The fractions were vacuum centrifuged to dryness and stored at −80°C until analysis by mass spectrometry.

#### Mass spectrometry acquisition and data analysis

The dried peptide fractions were reconstituted in 0.1% TFA and subjected to nanoflow liquid chromatography (Thermo Ultimate 3000RSLC Nano LC System, Thermo Scientific) coupled to an Orbitrap Eclipse mass spectrometer (Thermo Scientific). Peptides were separated using a low pH gradient using 5%–50% ACN over 120 min in mobile phase containing 0.1% formic acid at a 300 nL/min flow rate. MS scans were performed in the Orbitrap analyzer at a resolution of 120,000 with an ion accumulation target set at 4e^5^ and max IT set at 50 ms over a mass range of 400–1,600 m/z. Ions with determined charge states between 2 and 5 were selected for MS2 scans in the ion trap with CID fragmentation (Turbo; NCE 35%; maximum injection time 35 ms; AGC 1 × 10^4^). The spectra were searched using the Real Time Search node in the tune file using the human UniProt database using the Comet search algorithm with TMT16 plex (304.2071 Da) set as a static modification of lysine and the N-termini of the peptide. Carbamidomethylation of cysteine residues (+57.0214 Da) was set as a static modification, while oxidation of methionine residues (+15.9949 Da) was set up as a dynamic modification. For the selected peptide, an SPS–MS3 scan was performed using up to 10 *b*- and *y*-type fragment ions as precursors in an Orbitrap at 50,000 resolution with a normalized AGC set at 500, followed by a maximum injection time set as “auto” with a normalized collision energy setting of 65. Acquired MS/MS spectra were searched against a human UniProt protein database along with a contaminant protein database, using SEQUEST and Percolator validator algorithms in the Proteome Discoverer 2.4 software (Thermo Scientific). The precursor ion tolerance was set at 10 ppm, and the fragment ion tolerance was set at 0.02 Da along with methionine oxidation included as a dynamic modification. Carbamidomethylation of cysteine residues and TMT16-plex labeling (304.2071 Da) were set as static modifications of lysine residues and peptide N-termini. Trypsin was specified as the proteolytic enzyme, with up to two missed cleavage sites allowed. Searches used a reverse sequence decoy strategy to control for the false peptide discovery, and identifications were validated using percolator software. Reporter ion intensities were adjusted to correct for the impurities according to the manufacturer’s specifications, and the abundances of the proteins were quantified using the summation of the reporter ions for all identified peptides. The reporter abundances were normalized across all the channels to account for equal peptide loading.

### Proximity ligation assay

*In situ* PLA was performed with the Duolink *In Situ* Red Starter Kit Mouse/Rabbit (Sigma, DUO92101) according to the manufacturer’s protocol. Cells were seeded in eight-well Mu chamber slides (Ibidi, 80826) at 15,000 cells per well. For HEK293T cells, chamber slides were coated with 3 mg/mL PureCol for 1 h at room temperature and allowed to dry prior to cell seeding. Cells were fixed/permeabilized with Cytofix/Cytoperm Solution (BD, B554714) for 5 min and blocked with Duolink Blocking Solution (1×) for 1 h at 37°C. Cells were then incubated with primary antibodies (rabbit anti-IFITM3 [Abcam, ab109429] and mouse anti-IFITM1 [Proteintech, 60074-1-Ig]) for 1 h at room temperature. Cells were washed twice with buffer A and subsequently incubated with the probes’ affinity-purified donkey anti-rabbit IgG (Anti-Rabbit PLUS, Sigma; DUO92002) and affinity-purified donkey anti-mouse IgG (Anti-Mouse PLUS, Sigma; DUO92004) for 1 h at 37°C. After washing cells twice with buffer A, oligonucleotide ligation was performed for 30 min at 37°C. Cells were washed two additional times with buffer A, followed by incubation with amplification stock solution for 100 min at 37°C. After washing twice with buffer B, Hoechst 33,342 (Thermo Fisher, H3570) was added for 5 min at room temperature to label nuclei. Image acquisition was performed with a Leica Stellaris confocal fluorescence microscope.

### siRNA transfection

For immunofluorescence experiments, HeLa cells were transfected with 20 nM Silencer Select siRNA (Thermo Fisher) (negative control #1 [4390844]) or 20 nM IFITM3 (4392421, s195035) using Lipofectamine RNAiMAX according to the manufacturer’s instructions. siRNA and RNAiMAX were diluted in OptiMEM Reduced Serum Medium (Gibco). Cells were cultured at 37°C for 72 h post-transfection followed by fixation, permeabilization, and immunostaining for confocal immunofluorescence microscopy. For influenza A virus infection experiments, HeLa cells were transfected with 40 nM Silencer Select siRNA (Thermo Fisher) (negative control #1 [4390844]), 20 nM IFITM1^A^ (4392421, s194939), 20 nM IFITM1^B^ (4392421, s228411), 20 nM IFITM3 (4392421, s195035), or 20 nM IFITM1^A^ (4392421, s194939) or 20 nM IFITM1^B^ (4392421, s228411) plus 20 nM IFITM3 (4392421, s195035) using Lipofectamine RNAiMAX according to the manufacturer’s instructions. siRNA and RNAiMAX were diluted in OptiMEM Reduced Serum Medium (Gibco).

### Virus infection

HeLa cells were transfected with siRNA as described above and, 72 h later, inoculated with Influenza A/PR/8/34 (H1N1) (ATCC, VR-95) at an MOI of 0.1 for 18 h at 37°C. Cells were fixed and permeabilized with BD Cytofix/Cytoperm and immunostained with InVivoMab anti-Influenza A virus NP (BioXCell, BE0159). The percentage of NP + cells was measured with a BD LSRFortessa flow cytometer.

### Statistical analysis

Tests for statistical significance were performed in GraphPad Prism.

## Data Availability

All data supporting the conclusions of the study are included in this article.

## SUPPLEMENTAL MATERIAL

Data Set S1 jvi.00677-26-s0001.xlsx IP-MS data set.

Supplemental figures jvi.00677-26-s0002.pdf Fig. S1 and S2.

ASM does not own the copyrights to Supplemental Material that may be linked to, or accessed through, an article. The authors have granted ASM a non-exclusive, world-wide license to publish the Supplemental Material files. Please contact the corresponding author directly for reuse.
