# Supplementary material for: IFITM1 and IFITM3 cooperate to restrict virus entry in endolysosomes
Source: J Virol. 2026 Jun 9;100(7):e00677-26. doi: 10.1128/jvi.00677-26 (PMC13386968; doi:10.1128/jvi.00677-26)
Supplement: Supplemental figures — Fig. S1 and S2. [file jvi.00677-26-s0002.pdf]

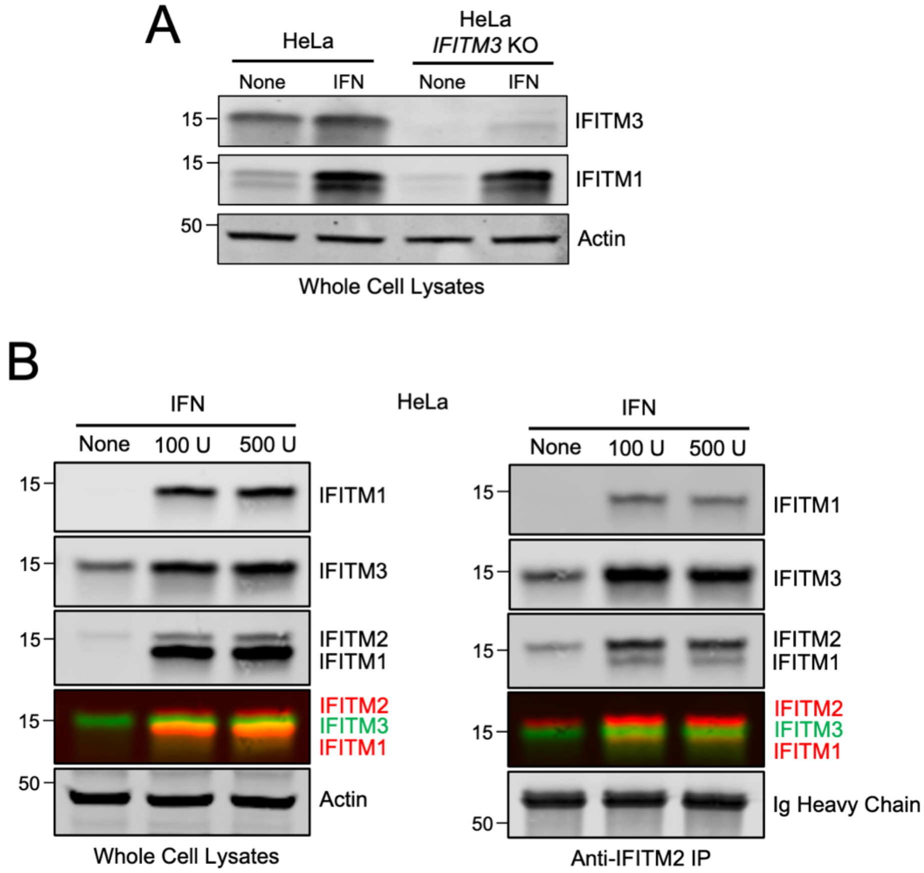

Supplemental Figure 1:
(A) HeLa and HeLa IFITM3 knockout cells were untreated or treated with 100 units IFNb for 18 hours and lysed. Whole cell lysates were subjected to SDS-PAGE and immunoblotting with anti-IFITM3, anti-IFITM1, and anti-Actin (used as loading control). (B) Left: HeLa cells were untreated or treated with 100 units or 500 units IFNb1a for 18 hours and lysed. Whole cell lysates were subjected to SDS-PAGE and immunoblotting with anti-IFITM1, anti-IFITM3, anti-IFITM2, and anti-Actin (used as loading control). Right: IFITM2 was immunoprecipitated with anti-IFITM2 and IP fractions were subjected to SDS-PAGE and immunoblotting with anti-IFITM1, anti-IFITM3, and anti-IFITM2 (immunoglobulin heavy chain was used as loading control). Numbers and tick marks left of blots indicate position and size (in kilodaltons) of protein standard in ladder. All immunoblots were performed twice, and one representative example is shown.

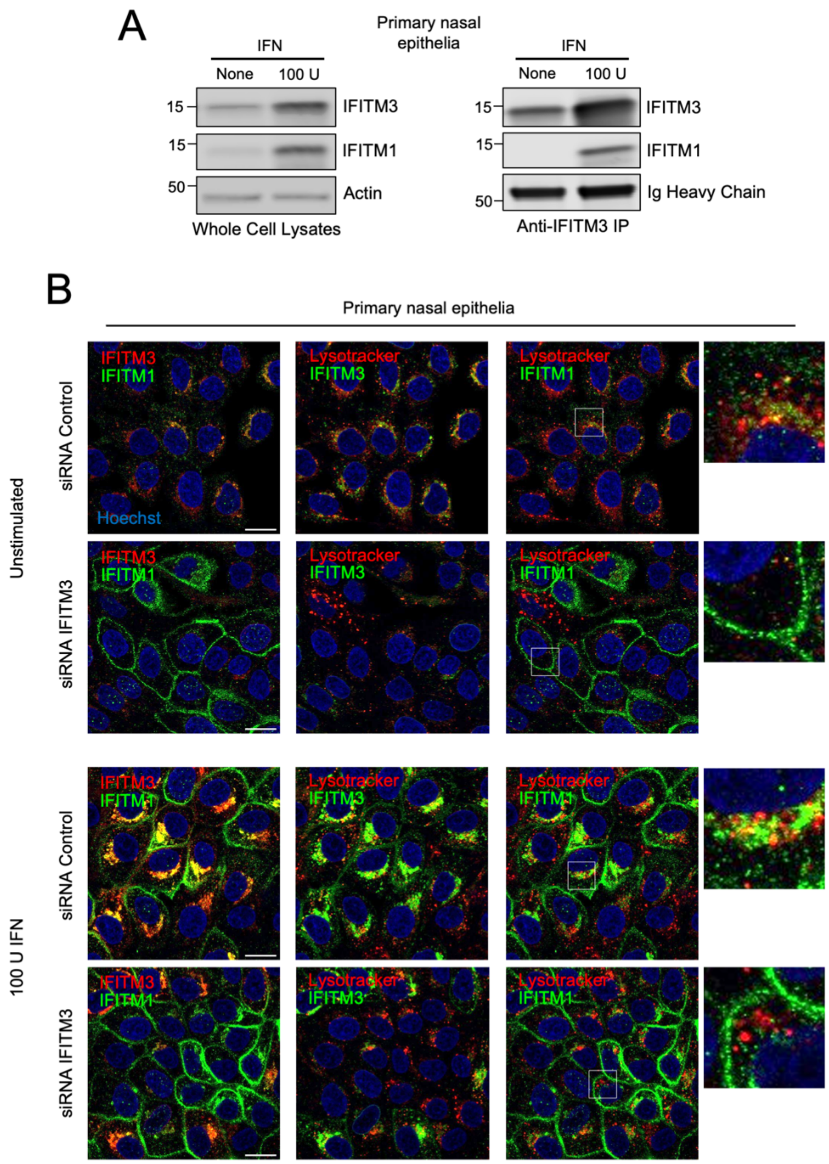

Supplemental Figure 2:

(A) Left: primary human nasal epithelial cells were untreated or treated with 100 units IFNb for 18 hours and whole cell lysates were subjected to SDS-PAGE and immunoblotting with anti-IFITM3, anti-IFITM1, and anti-Actin (used as loading control). Right: IFITM3 was immunoprecipitated with anti-IFITM3 and IP fractions were subjected to SDS-PAGE and immunoblotting with anti-IFITM3 and anti-IFITM1 (immunoglobulin heavy chain was used as loading control). Numbers and tick marks left of blots indicate position and size (in kilodaltons) of protein standard in ladder. (B) Primary human nasal epithelial cells were transfected with control siRNA or siRNA targeting IFITM3 for 48 hours and subsequently treated with 100 units IFNb1a for 18 hours or left untreated. Cells were stained with Lysotracker, fixed and permeabilized, and immunostained with anti-IFITM1 and anti-IFITM3 followed by confocal immunofluorescence microscopy. Nuclei were labeled with Hoechst. Scale bar = 15 microns. All immunoblots and microscopy experiments were performed twice, and one representative example is shown.
